# Nitric Oxide Pre-Treatment Advances Seed Germination and Alleviates Copper-Induced Photosynthetic Inhibition in Indian Mustard

**DOI:** 10.3390/plants9060776

**Published:** 2020-06-20

**Authors:** Bilal A. Rather, Iqbal R. Mir, Asim Masood, Naser A. Anjum, Nafees A. Khan

**Affiliations:** Plant Physiology and Biochemistry Laboratory, Department of Botany, Aligarh Muslim University, Aligarh 202002, India; saffibilal@gmail.com (B.A.R.); m3riqbal@gmail.com (I.R.M.); anjum@ua.pt (N.A.A.); naf9.amu@gmail.com (N.A.K.)

**Keywords:** copper stress, nitric oxide pre-treatment, germination rate, photosynthesis

## Abstract

This investigation tested the efficiency of nitric oxide (NO) in alleviation of Cu-induced adverse impacts on seed germination and photosynthesis in Indian mustard (*Brassica juncea* L.). Pre-treatment of *B. juncea* seeds with sodium nitroprusside (SNP; NO donor) significantly improved the seed germination rate and also alleviated Cu-accrued oxidative stress. However, in the absence of NO, Cu caused a higher reduction in seed germination rate. The presence of NO strengthened the antioxidant defense system (glutathione reductase, ascorbate peroxidase, and superoxide dismutase) and thereby sustained the lower lipid peroxidation, reduced H_2_O_2_ content, and thiobarbituric acid reactive substances in Cu-exposed seeds. NO pre-treated seeds also retained a higher amylase activity and exhibited an improved seed germination rate. This effect of NO under Cu stress was also seen in plants originated from the NO pre-treated seeds, where the role of NO pre-treatment was reflected in the improved photosynthetic potential of *B. juncea*. Overall, NO pre-treatment not only improved the germination rate in seeds but also carried its effects in the grown seedlings evidenced as improved photosynthesis and growth. Potential mechanisms involved in the action of NO pre-treatment included NO-mediated significant strengthening of the antioxidant defense system and decreases in Cu-caused oxidative stress parameters.

## 1. Introduction

Studies on heavy metal accumulation and its continuous addition in the environment affecting the agricultural system and human health have been known for a long time especially in less developed countries [1,2]. In this perspective, Cu has emerged as a severe pollutant because of its extensive use in industries and as a pesticide in the agricultural system [3,4]. Based on its available concentration, Cu performs a remarkable array of functions in plants. Generally, Cu is widely accepted as an essential micronutrient element for plants [4]. Proteins containing Cu as a cofactor take part in various biochemical processes, involved in plant growth and development, and protective mechanisms [5]. The optimum levels of Cu in leaves are established as 10 µg g^−1^ dry mass [6]. However, the acute levels of toxicity found for most of the crop plants are slightly higher (20–30 µg g^−1^ dry mass) [7]. Indian mustard (*Brassica juncea* L.) being the hyperaccumulator of metal(oids) can accumulate a high concentration of Cu and translocate much into above-ground parts that can be detrimental to any plant growth. Thus, maintaining Cu-homeostasis is essential for a plant to survive under high Cu, either through vacuolar sequestration, storage, or transport of metal ions from the cytoplasm to outer compartments [8,9,10]. This ability of *B. juncea* makes it a suitable model plant material to study the impact of high Cu concentration.

Photosynthetic functions are essentially influenced by the presence of a high Cu level. An elevated Cu level in leaves damages thylakoids; dissipates the electron transport in photosystem (PS) II (PSII), alters photophosphorylation and membrane integrity; and eventually decreases photosynthesis and major gas-exchange parameters [11,12,13]. Additionally, high concentrations of Cu induce oxidative stress that causes membrane disruption and lipid peroxidation [14,15]. Moreover, excess Cu also alters the process of mineral uptake [16,17] and also retards the seed germination process and overall health of the developing seedling [4]. Further, excess Cu stimulates the activity of phytochelatin synthase and increases the production of phytochelatins (PCs), cysteine-rich metal-chelating peptides. The production of PCs results in the chelation of Cu by sequestering the PCs–Cu conjugant in vacuoles decreasing free Cu concentration. Since PCs are oligomers of reduced glutathione (GSH), this can deplete the pool of GSH, an important metabolite involved in the maintenance of the cellular redox balance [18,19]. Thus, evaluating the potential influence of Cu on seed germination is a prerequisite for assessing its effects on various plant developmental stages. The studies have well documented the deleterious effects of Cu on seed germination and associated metabolic processes [4,20,21,22,23,24,25]. Copper toxicity is known to alter metabolism during seed germination and also to interfere with the pathway of ubiquitin-proteasome mechanism to oxidatively damaged proteins [20]. Elevated Cu concentrations were reported to inhibit the radicle elongation rate in *Alyssum montanum* and *Thlaspi ochroleucum* [21].

Modulation of the signaling of phytohormones has been used as a potential tool to enhance the adaptability of plants under abiotic stress conditions. In particular, nitric oxide (NO), a versatile signaling molecule has been found to play critical roles in plant defense reactions including induction of antioxidant potential under various plant abiotic stresses [26,27]. Adequate evidence substantiates a wide range of roles of NO in plant physiological processes including germination, photosynthesis, and defense mechanism both under normal and environmental stresses [28,29]. In plants, the cell-protective role of NO has been tested with DNA, proteins, lipids, and chlorophyll [30]. Sorghum seeds were reported to exhibit NO-production during germination [31]. Treatment of sodium nitroprusside (SNP; NO-source) found to enhance seed germination and also to break seed dormancy [32,33,34,35]. Moreover, NO-treatment promoted seed germination of wheat under elevated Cu-levels by improving antioxidant capacity [36]. Nitric oxide was also found to play a prominent role in the regulation of S-assimilation [37,38,39]. The reported involvement of NO in S-assimilation has been argued to result in a higher rate of S-assimilation yielding more cysteine and GSH production, and thereby maintaining PCs level optimum for detoxifying high metal ions [40,41].

In the present reported research, the effect of Cu-toxicity and the significance of NO in averting this toxicity in *B. juncea* were studied. The role of NO pre-treatment to seeds was studied in the alleviation of Cu-induced inhibition of seed germination in terms of measuring germination rate and amylase activity. Further, it was also investigated if this pre-treatment of NO could carry its effects to influence the antioxidant metabolism, photosynthetic functions, and growth at maturity without supplementation of NO at later developmental stages of plant growth. Modulation of the antioxidant system has always been proved as a strategy for improving stress impact tolerance mechanisms in plants via improved photosynthetic potential.

## 2. Materials and Methods

### 2.1. Treatments

Healthy uniform seeds of Indian mustard (*Brassica juncea* L. cv. Pusa Tarak) were surface sterilized with 0.1% hypochlorite (*v*/*v*) solution and washed several times with autoclaved double distilled water. The sterilized seeds were randomly placed on two filter papers covered Petri-plates for germination at 28 ± 2 °C containing 0, 0.5, 1, 2, 3, 4, 5, 6, 7, 8, 9, and 10 mM copper sulphate (CuSO_4_) for 3 days. Fifty seeds were placed on each Petri plate (*n* = 50). Standard radicle emergence of seeds was scored as the germination percentage. Since, 3.0 mM Cu concentration reduced the germination percentage near to half of the control, this concentration was considered as semi-lethal (LD_50_).

To understand NO action on seed germination under Cu stress, seeds of *B. juncea* were pre-treated with 0, 25, 50, 100, 200, and 250 μM SNP (NO donor) for 3 h, and later exposed to semi-lethal 3.0 mM Cu for 3 days. The screening of SNP concentration was done to find out its optimal concentration that can efficiently alleviate Cu-inhibited seed germination. Sodium nitroprusside SNP at 100 μM proved most effective in reducing 3.0 mM Cu and was used for further experimentations. In further experiments, the influence of NO action in presence of Cu was confirmed using its analogue potassium ferrocyanide (FCy) and its scavenger 2-(4-carboxyphenyl)-4,4,5,5-tetramethylimidazoline-1-oxyl-3-oxide (cPTIO). The treatment included control (seeds germinated in deionized water only), 100 μM SNP (optimum concentration), 100 μM (FCy) or 100 μM SNP plus 200 μM cPTIO (SNP + cPTIO) for 3 h, and later exposed to the semi-lethal Cu concentration (3.0 mM).

In another experiment, the influence of NO action was studied on photosynthesis, growth and antioxidant metabolism at 30 days after germination (DAG). Another aim of this experiment was also to ascertain the effect of NO-mediated alleviation of Cu’s impacts on seed germination in developing plants. For this, *B. juncea* seeds were washed and surface-sterilized with 0.1% hypochlorite (*v*/*v*) and were germinated in pots filled with acid-washed autoclaved sand. The plants were raised in modified Hoagland nutrient solution applied on alternate days consisting of 3.0 mM KNO_3_, 0.5 μM CuSO_4_, 2.0 mM Ca(NO_3_)_2_, NH_4_H_3_ PO_4_, 25 μM H_3_BO_4_, 25 μM H_3_BO_4_, 2.0 μM MnCl_2_, 50 μM KCl, 20 μM ZnSO_4_, 20 μM Na_2_Fe-EDTA and 0.5 μM (NH_4_)_6_Mo_7_O_24_. The pots were placed in a naturally illuminated net house with an average temperature of 22 ± 3 °C during day and 14 ± 2 °C at night. In this experiment, seeds were pre-treated (before sowing) for 3 h with deionized water, 100 μM SNP, 100 μM FCy, or 100 μM SNP plus 200 μM cPTIO and were sown in pots in the presence or absence of 3.0 mM Cu in the form of CuSO_4_. The experimental design is given in Table 1. The treatments were arranged in a randomly blocked design with three replicates (*n* = 3) for each treatment. Plants were sampled at 30 DAG for recording different parameters.

### 2.2. Determination of H_2_O_2_ Content and Lipid Peroxidation

The assay of H_2_O_2_ was done following the method of Okuda et al. [42]. Germinated seeds (0.5 g) and fresh leaves (0.5 g) of the two experiments were ground in ice-cold 200 mM perchloric acid. After centrifugation at 1200× *g* for 10 min, perchloric acid of the supernatant was neutralized with 4 M KOH. The insoluble potassium perchlorate was eliminated by centrifugation at 500× *g* for 3 min. The reaction was started by the addition of peroxidase and an increase in the absorbance was recorded at A_590_ for 3 min. Contents of TBARS were measured according to Dhindsa et al. [43] by recording absorbance at 532 nm. Values obtained were corrected for non-specific turbidity by subtracting the absorbance at 600 nm. The TBARS content was calculated using its extinction coefficient of 155 mM^−1^ cm^−1^.

### 2.3. Assay of Antioxidant Enzymes

Germinated seeds (0.5 g) and fresh leaves (0.2 g) of the two experiments were homogenized with mortar and pestle using 100 mM potassium phosphate buffer (pH 7.0) containing 1% polyvinylpyrrolidone (PVP) (*w*/*v*) and 0.05% Triton X-100 (*v*/*v*). Later the centrifugation of homogenized material was done at 15,000× *g* for 20 min. The supernatant generated after centrifugation was utilized to assay the activity of glutathione reductase (GR) and superoxide dismutase (SOD). The assay of ascorbate peroxidase (APX) required addition of extraction buffer supplemented with 2.0 mM ascorbate.

Activity of SOD was determined as per the protocol of Beyer and Fridovich [44], and Giannopolitis and Ries [45] by monitoring the inhibition of photochemical reduction of nitro blue tetrazolium (NBT). Reaction mixture (5.0 mL) consisted of 50 mM Na_2_CO_3_ (pH 10.0), 5.0 mM HEPES (pH 7.6), 0.1 mM EDTA, 0.025% (*v*/*v*) Triton X-100, 13 mM methionine, 63 mM NBT and 1.3 mM of riboflavin. The extract of enzyme was illuminated for 15 min (360 μmol m^2^ s^−1^), and a set without illumination acted as a control to correct the turbidity of background absorbance. A unit of enzyme was expressed as the amount of the enzyme that inhibited the NBT reduction by 50% at 560 nm. The amount of the enzyme that inhibited the reduction of NBT by 50% at 560 nm was equal to one unit of SOD. Activity of APX and GR was assessed as per the protocol adopted by Nakano and Asada [46] and Foyer and Halliwell [47]. The detailed procedure for determining APX and GR activity was explained in our earlier study [41].

### 2.4. Assay of Amylase Activity

Seeds were pre-treated with water or with SNP (100 μM). Later, pre-treated seeds were kept for germination for 48 h in Petri plates containing 3 mM CuSO_4_ solution. Seeds (0.5 g) were then collected and homogenized in a pre-chilled mortar and pestle with 6 mL of 50 mM Tris-HCl (pH 7.5) containing 1% PVP and 15 mM 2-mercaptoethanol. The homogenate was collected and centrifuged at 4 °C at 10,000× *g* for 30 min. After centrifugation, the resulting supernatant was used for determining enzyme activity. The amylase activity was determined by using the protocol of starch-iodine following Collins et al. [48]. A unit of enzyme was expressed by taking the enzyme quantity to reach 50% of the original color intensity.

### 2.5. Determination of GSH Content

The GSH content in fresh leaves was determined spectrophotometrically according to the protocol provided by Anderson [49]. Using pre-chilled mortar and pestle, under 4 °C fresh leaf tissue (0.5 g) was crushed in 2.0 mL of 5% sulphosalicylic acid. Centrifugation of homogenized material was done at 10,000× *g* for 10 min. Then, 0.6 mL of phosphate buffer (100 mM, pH 7.0) and 40 mL of 5,5′-dithiobis-2-nitrobenzoic acid were added to the 0.5 mL of supernatant. After 2 min, the absorbance was recorded at 412 nm. The detailed protocol has been given in our earlier publication [50].

### 2.6. Histochemical Detection of Reactive Oxygen Species

Histochemical staining of seed and leaf samples was performed using the protocol given by Kumar et al. [51]. Nitro blue tetrazolium (NBT) and 3,3-diaminobenzidine (DAB) were used for the assay accumulation of both superoxide ion (O_2_^−^) and H_2_O_2_ in seed and leaf samples of different treatments. The samples from each treatment were kept in NBT solution prepared by dissolving 0.1 g of NBT in 50 mL of 50 mM sodium phosphate buffer (pH 7.5) in an amber-colored bottle and were incubated overnight. The stained samples were immersed in absolute ethanol and boiled in water-bath for 10 min for discoloration to get the staining clear. For DAB staining solution, 50 mg DAB was dissolved in 50 mL double-distilled water in an amber-colored bottle with pH 3.8. The samples from each treatment were incubated for 8 h in DAB solution. Subsequently, the stained samples were immersed in absolute ethanol and boiled in a water bath for 10 min for discoloration to get a clear visualization of the stained samples.

### 2.7. Confocal Laser Microscopy Study for ROS Imaging and Cell Viability Determination

For ROS imaging, root samples 1–2 cm in length were dipped into the 12.5 μM 2′,7′ dichlorofluorescin diacetate (H2DCFDA) solution in a Petri dish for 15 min and washed 3 times properly with double distilled water. Stained samples were kept on a glass slide and studied under a confocal microscope (excitation 400–490 nm, emission ≥ 520 nm). For the cell viability test, properly washed root samples (1–2 cm length) of each treatment were dipped into 25 μM propidium iodide (PI) solution. Stained samples were washed appropriately, placed on a glass slide, and were observed under a confocal microscope.

### 2.8. Determination of Cu Concentration

Leaf and root samples were dried in the oven for two days at 80 °C. The oven-dried samples were crushed to a fine powder in mortar and pestle. This fine powder was digested with a solution containing concentrated HNO_3_/HClO_4_ (3:1, *v*/*v*) and was diluted with water. Concentration of Cu was determined by Atomic Absorption Spectrophotometer (GBC, 932 plus; GBC Scientific Instruments, Braeside, Australia).

### 2.9. Photosynthetic Characteristics

Net photosynthesis (P_N_), intercellular CO_2_ (C_i_) and stomatal conductance (g_S_) were measured in second topmost leaves of plants using Infrared gas analyzer (CID-340, Photosynthesis System, Bio-Science, Washington, USA). These parameters were recorded between 11 am to 12 noon when the photosynthetically active radiation (PAR) was above 780 μmol m^−2^ s^−1^ and at 380 ± 5 μmol^−1^ atmospheric CO_2_ concentrations.

Maximal quantum efficiency of photosystem II (PSII) (*F_v_*/*F_m_*) of full-fledged leaf second from the top was noted with the help of chlorophyll fluorometer (JUNIOR PAM, Heinz Walz, Germany). Proceeding to get the result of maximum fluorescence (*F_m_*) and minimal fluorescence (*F_o_*) intensity, leaf samples were kept in the dark condition for 30 min. Weak measuring pulses (0.1 µmol m^−2^ s^−1^) and saturating pulse (>6000 µmol m^−2^ s^−1^) were used to measure *F_o_* and *F_m_*, respectively. Difference between *F_o_* and *F_m_* was used to calculate the variable fluorescence (*F_v_*). The maximum quantum yield efficiency of PS II was calculated as a ratio of *F_v_* to *F_m_.*

Activity of Rubisco in leaves was monitored by adopting the procedure of Usuda [52]. Fresh leaf samples (1.0 g) were ground in a pre-chilled mortar and pestle with an extraction buffer containing 0.25 M Tris-HCl (pH 7.8), 0.0025 M EDTA, 0.05 M MgCl_2_ and 37.5 mg dithiothreitol (DTT). Centrifugation of homogenized material was done at 10,000× *g* for 10 min at 4 °C. The resulting supernatant brought after centrifugation was used to measure enzyme activity. Reaction mixture (3.0 mL) contained 100 mM Tris-HCl (pH 8.0), 10 mM MgCl_2_, 0.2 mM NADH, 40 mM NaHCO_3_, 5.0 mM DTT, 4 mM ATP, 1U of 3-phosphoglycerate kinase, 1U of glyceraldehyde 3-phosphodehydrogenase, and 0.2 mM ribulose 1,5-bisphosphate (RuBP). Bradford [53] method was adopted to estimate protein content.

### 2.10. Determination of Growth Parameters

The plant samples from each treatment were dried in a hot-air oven at 80 °C. The dried leaf samples were weighed on an electrical balance and the weight was recorded as whole plant dry mass. To measure leaf area, leaf area meter (LA 211, Systronics, New Delhi, India) was used.

### 2.11. Physiological Measurements of Guard Cells

Upper leaves of 30 days old plants were plugged from each plant with different treatments and were fixed by 2.5% glutaraldehyde, and stomatal images were taken using scanning electron microscopy (JSM-6510 LV, JEOL, Tokyo, Japan). The epidermal peels of the leaf samples were removed from the abaxial side and stomatal images of the sections at 40× were taken using a compound microscope outfitted with NIKON digital camera. The stomatal aperture width was measured with the help of a micrometer scale.

### 2.12. NO Generation

The level of NO generation was confirmed by estimating nitrite content adopting the protocol given by Zhou et al. [54] with slight modifications. Using pre-chilled mortar and pestle, leaf samples (500 mg) were ground in 3.0 mL of 50 mM ice-cold acetic acid buffer (pH 3.6) containing 4% zinc acetate; and later, was centrifuged at 11,500× *g* for 15 min at 4 °C. The pellet obtained was washed twice with 1.0 mL of the extraction buffer and then centrifuged again. The resulting supernatants from the two spin were mixed and neutralized by the addition of 100 mg of charcoal. After brief vortex, the filtrate was collected. Each filtrate of 1.0 mL and Greiss reagent (0.1% *N*-1-naphthyl ethylenediamine dihydrochloride and 1% sulphanilamide in 5% H_2_PO_4_ solution) were mixed in the ratio of (1:1) and then for 30 min these were incubated at room temperature. The absorbance was read at 540 nm and NO content was measured from a calibration curve plotted using sodium nitrite as standard.

### 2.13. Statistical Analysis

Data obtained in the experiments were analyzed statistically using analysis of variance (ANOVA) by SPSS 17.0 for Windows and presented as treatment mean ± SE. Treatment means were compared using the least significant difference (LSD) at *p* < 0.05. Bars with the same letter are not significantly different by LSD test at *p* < 0.05.

## 3. Results

### 3.1. Effect of Cu Stress on Seed Germination Percentage

**Figure 1:**
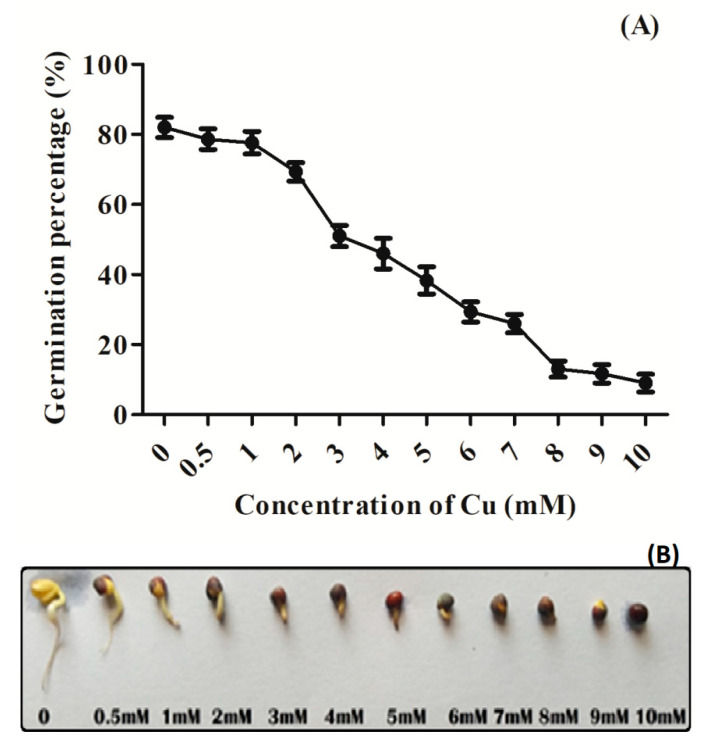
(**A**) Germination percentage; (**B**) phenotype of randomly selected germinating seeds of Indian mustard (*Brassica juncea* L.) exposed to copper (Cu) concentrations (0, 0.5, 1, 2, 3, 4, 5, 6, 7, 8, 9 and 10 mM) for 3 days.

The germination percentage of seeds declined with the increasing Cu concentration from 1 to 10 mM (Figure 1A). At 3.0 mM Cu concentration, germination percentage declined nearly half of the control and was marked as a semi-lethal concentration and was selected for further experimentations. Figure 1B offers an overview showing Cu-induced inhibitory effect on seed germination under various Cu concentrations.

### 3.2. Effect of NO Pre-treatment on Germination of Cu Stressed Seeds

**Figure 2:**
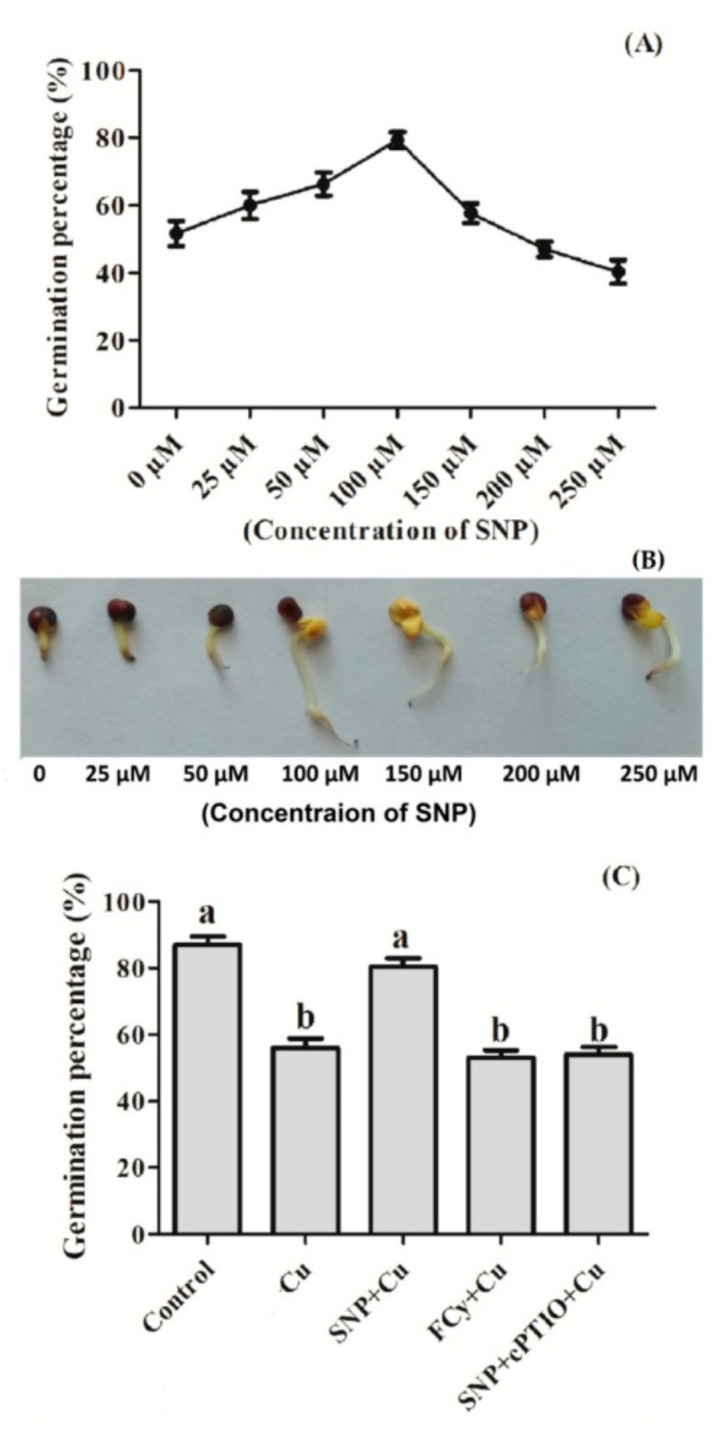
(**A**) Germination percentage under varying concentration of SNP under Cu stress; (**B**) phenotype of randomly selected germinating seeds of Indian mustard (*Brassica juncea* L.) pre-treated with 0, 25, 50, 100, 150, 200, and 250 µM SNP and exposed to 3.0 mM Cu; and (**C**) germination percentage in *B. juncea* seeds pre-treated with water, SNP, FCy (SNP analogue) and SNP + cPTIO (cPTIO as a specific NO-scavenger), later exposed to 3.0 mM Cu for 3 days. Same letter above bars show that data did not differ significantly by LSD test at *p* < 0.05.

Seeds pre-treated with 0, 25, 50, 100, 200, and 250 µM SNP concentration for 3 h were later exposed to 3.0 mM Cu showed increased germination percentage up to 100 µM SNP treatment, then dropped to approximately the same as control at 250 µM. Here, 100 µM SNP was proved to be the most effective treatment against 3.0 mM Cu stress-induced inhibition of seed germination (Figure 2A). Figure 2B is a critique of seed germination under varying SNP concentrations. On the other hand, pre-treatment with SNP showed an increase in seed germination percentage under Cu stress. But the pre-treatment of Cu exposed seeds to NO analogue FCy showed no significant change in germination percentage in comparison to Cu exposed seeds. Further, the use of cPTIO along with SNP in Cu exposed seeds reversed the SNP induced inhibition of Cu stress effect on seed germination (Figure 2C).

### 3.3. Effect of NO on H_2_O_2_ and TBARS Contents in Germinating Seeds under Cu Stress

**Figure 3:**
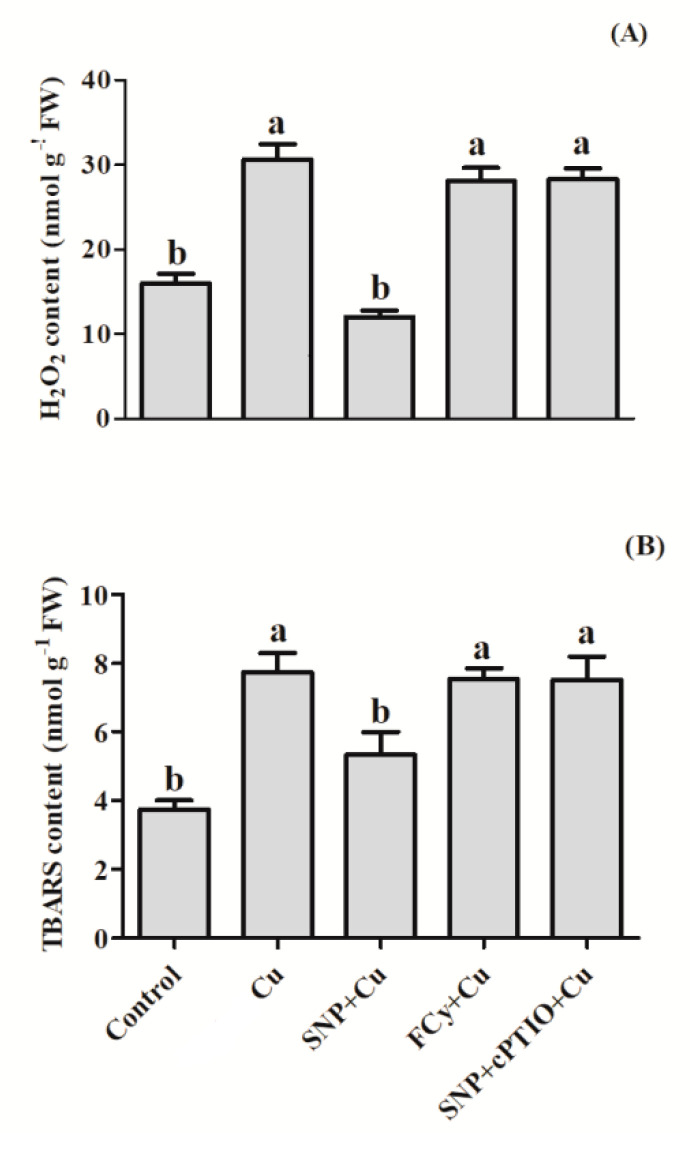
H_2_O_2_ (**A**) and TBARS (**B**) content in germinating seeds of Indian mustard (*Brassica juncea* L.). Seeds were germinated with/without Cu stress and pre-treated with water, SNP, FCy (SNP analogue) and SNP + cPTIO (cPTIO as a specific NO-scavenger), later exposed to 3.0 mM Cu stress for 3 days. Same letters above bars show that data did not differ significantly by LSD test at *p* < 0.05. FW—fresh weight.

Copper stress markedly increased the content of H_2_O_2_ and TBARS where the values increased by 152.7 % and 106.7% compared to that of control seeds. On the other hand, application of SNP (prior germination) substantially reduced H_2_O_2_ by 33.1% and TBARS by 42.6% over the untreated control seeds. In addition, pre-germination application of FCy or SNP in combination with cPTIO in Cu-stressed seeds showed very similar result with stressed seeds (water pre-treated) over the control (Figure 3).

Further SNP pre-treatment under Cu stress reduced H_2_O_2_ content by about 47.3% and TBARS 31% in comparison to the Cu-stressed seeds. However, FCy or SNP in combination with cPTIO pre-treatment reduced H_2_O_2_ content by 7.6% and 6.8%, respectively, as compared to Cu-stressed seeds. In the case of TBARS content, FCy or SNP in combination with cPTIO reduced it equally by 2.5% and 2.6% respectively in comparison to stressed seeds (Figure 3A,B). These results of FCy or SNP in combination with cPTIO were comparable to Cu-stressed seeds.

### 3.4. Effect of NO Application on Activities of Antioxidant Enzymes under Cu Stress during Seed Germination

**Figure 4:**
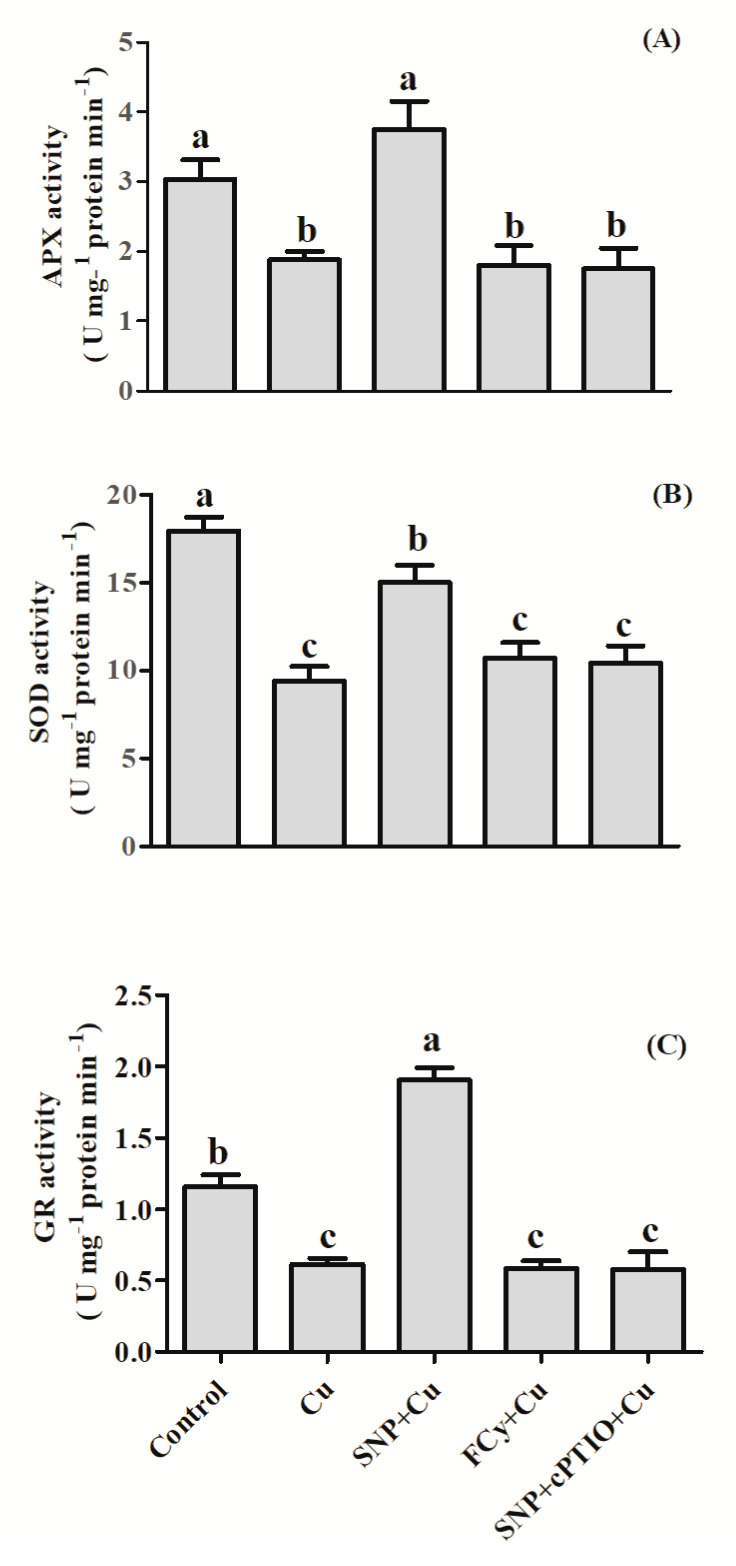
Activity of APX (**A**), SOD (**B**), and GR (**C**) in germinating seeds of Indian mustard (*Brassica juncea* L.). Seeds were germinated with/without Cu stress and pre-treated with water, SNP, FCy (SNP analogue) and SNP + cPTIO (cPTIO as a specific NO-scavenger), later exposed to 3.0 mM Cu stress for 3 days. Same letters above bars show that data did not differ significantly by LSD test at *p* < 0.05.

The performances of enzymatic antioxidants, such as APX, SOD, and GR, in mustard seeds are depicted in Figure 4. Results showed that activity of APX, GR and SOD were notably decreased in Cu-stressed seeds. In the presence of Cu, SNP did not influence activity of APX but significantly increased GR activity over the unstressed control seeds. On the other hand, SOD activity was lower in SNP treated seeds than those of control. However, the influence of FCy or SNP in combination with cPTIO showed results similar to stressed seeds.

Application of SNP with Cu boosted the activity of antioxidant enzymes to a higher level than Cu-stressed seeds, increasing the activity of APX by about 99.5%, SOD by about 59.6%, and GR by 210.3% in contrast to the stressed seeds. However, in the presence of Cu, SNP in combination with cPTIO showed no difference than the Cu-stressed seeds in the activities of the above enzymes. Similarly, FCy also showed significantly similar activity of APX, SOD, and GR as was under Cu treatment alone (Figure 4).

### 3.5. Effect of NO Application on ROS Accumulation in Germinating Seeds

**Figure 5:**
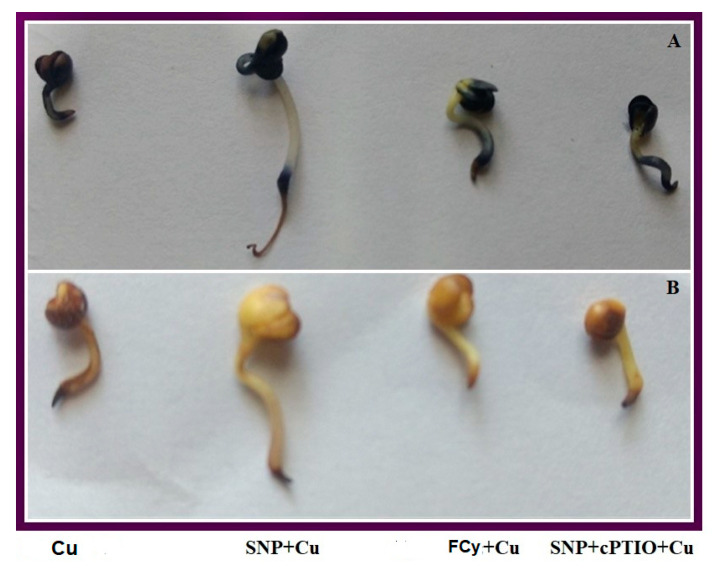
Accumulation of superoxide ion (O_2_^−^) using NBT staining, and (**A**) H_2_O_2_ using DAB staining (**B**) in germinating seeds of Indian mustard (*Brassica juncea* L.) pre-treated with water, SNP, FCy (SNP analogue) or SNP + cPTIO (cPTIO as a specific NO-scavenger), exposed to 3.0 mM Cu for 3 days.

Under Cu stressed conditions, seeds pre-treated with water, FCy or SNP in combination with cPTIO showed an increased O_2_^−^ accumulation as compared to SNP pre-treated seeds, which was observed by histochemical staining with NBT, showed dark blue stained area on radicle (a marker for O_2_^−^ ion accumulation). While H_2_O_2_ accumulation revealed using DAB staining showed more reddish-brown precipitate on water pre-treated seeds, FCy or SNP + cPTIO pre-treated seeds as compared to SNP treatment under Cu stress (Figure 5A,B).

### 3.6. Effect of NO Application on the Activity of Amylase

**Figure 6:**
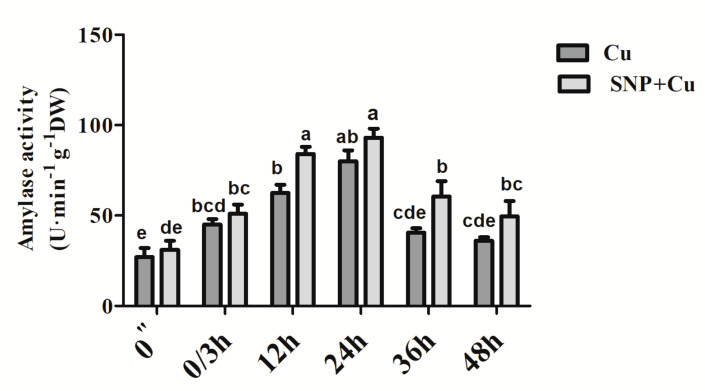
Activity of amylase in germinating seeds of Indian mustard (*Brassica juncea* L.) pre-treated with water or 100 µM SNP for 3 h (shown as from 0″ to 0/3 h of the treatment times) prior to exposing to 3.0 mM Cu for further 48 h (shown as 3/0, 12, 24, 36 and 48 h respectively). Same letters above bars show that data did not differ significantly by LSD test at *p* < 0.05. DW—dry weight.

From Figure 6, it can be observed that the activity of amylase increased substantially by the application of SNP to Cu-treated seeds compared to the seeds grown under Cu-stress. However, the optimum increase was noted at 12 and 24 h duration of SNP treatment, and then showed a reduction from 36 to 48 h duration.

### 3.7. Effect of NO Application on Cu Uptake and Levels of H_2_O_2_ and TBARS

**Figure 7:**
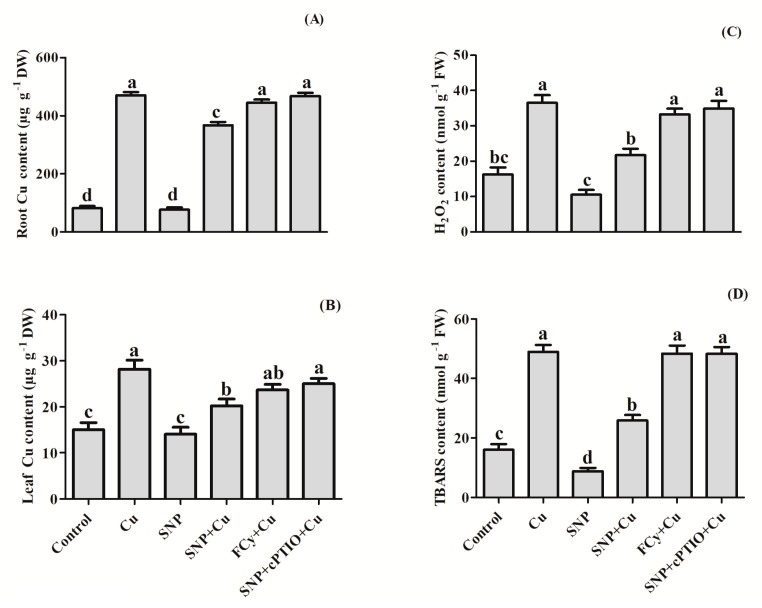
Contents of Cu in root (**A**) and leaf (**B**), and contents of both H_2_O_2_ (**C**) and TBARS (**D**) in leaves of Indian mustard (*Brassica juncea* L.) at 30 DAG. Plants were grown with/without Cu stress and treated during pre-germination for 3 h with water, SNP, FCy (SNP analogue) and SNP + cPTIO (cPTIO as a specific NO-scavenger). Same letters above bars show that data did not differ significantly by LSD test at *p* < 0.05. FW, fresh weight; DW, dry weight.

To establish the role of NO in the Cu accumulation in Cu-exposed *B. juncea* plants, Cu content was measured in both roots and leaves (Figure 7A,B). The Cu accumulation was found much higher in roots (470 µg g^−1^) than in leaves (28.02 µg g^−1^). Pre-germination application of SNP (NO donor) resulted in a decrease in Cu content level in both roots and leaves of Cu treated plants. However, the NO-mediated diminished accumulation of Cu was reversed by NO-scavenger, cPTIO when applied in combination with SNP. Similarly, FCy treatment showed a little or no significant reversal in Cu accumulation by plants.

Compared to control, plants receiving Cu showed greater content of H_2_O_2_ and TBARS (Figure 7C,D). The lone application of SNP exhibited decrease in H_2_O_2_ and TBARS content by about 35% and 45% without Cu stress. However, under Cu stress, this decrease in H_2_O_2_ and TBARS content was about 40% and 47% in comparison to Cu-treated plants. The protective effect of NO application was reversed by addition of cPTIO in Cu-fed plants. Further, FCy effect was significantly similar to the effect generated on application of cPTIO to SNP- and Cu-treated plants.

### 3.8. Effect of NO Treatment on ROS Accumulation by Leaves

**Figure 8:**
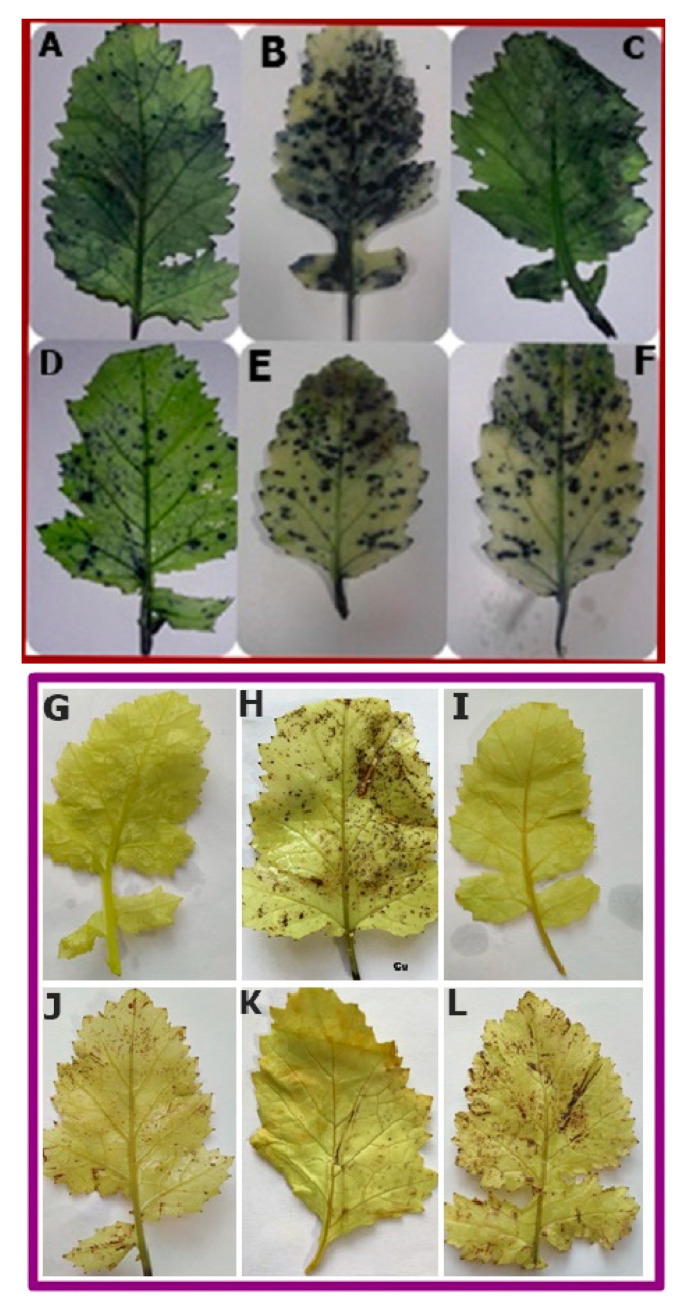
Accumulation of superoxide ion (O_2_^−^) and H_2_O_2_ in leaves of Indian mustard (*Brassica juncea* L.) stained with NBT (**A**–**F**) and DAB (**G**–**L**) respectively at 30 DAG. Plants were grown with/without Cu stress and treated during pre-germination for 3 h with water, SNP, FCy (SNP analogue) and SNP + cPTIO (cPTIO as a specific NO-scavenger). Control: **A** and **G**; **B** and **H**: Cu; **C** and **I**: SNP; **D** and **J**: SNP + Cu; **E** and **K**: FCy + Cu; **F** and **L**: SNP + cPTIO + Cu.

Both H_2_O_2_ and O_2_^−^ markedly accumulated in Cu-stressed plant (Figure 8A–F,G–L, represent H_2_O_2_ and O_2_^−^ accumulation respectively). Accumulation of H_2_O_2_ and O_2_^−^ was shown by histochemical staining with DAB and NBT, respectively. The leaves from the Cu-treated plants, with FCy and cPTIO in combination with NO applied plants showed discrete and deepest blue staining, which is a marker of O_2_^−^ accumulation. Addition of SNP to non-stressed seeds seemed closer to control in terms of staining markers. However, application of NO noticeably diminished ROS accumulation in the leaves of Cu-stressed *B. juncea* leaves. A similar result was observed in DAB staining; but in this case, brownish patches were observed which was a marker of H_2_O_2_ accumulation in leaves, showing NO induced reduction in H_2_O_2_.

### 3.9. Confocal Laser Scanning Microscopy

**Figure 9:**
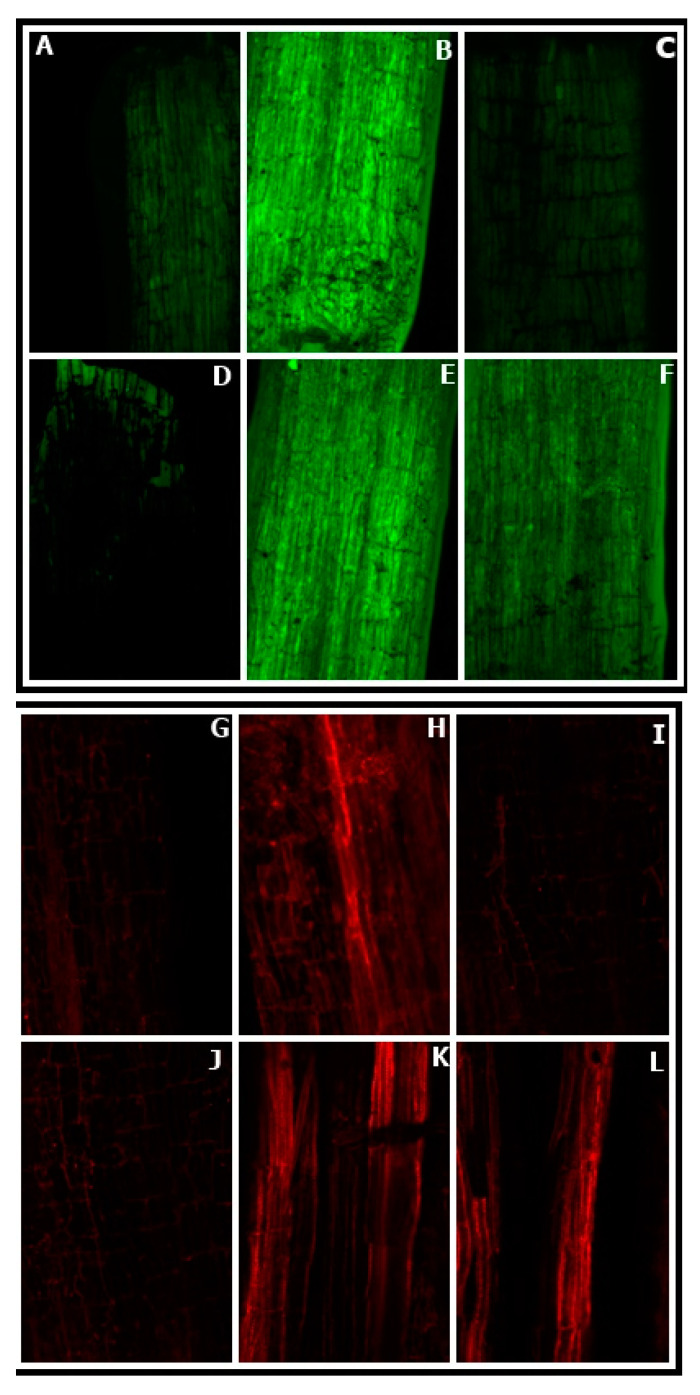
Confocal microscopic images of H_2_O_2_ formation in roots using H2DCFDA staining (**A–F**) and cell viability test (**G–L**) by propidium iodide staining, was performed on 30 days old roots of Indian mustard (*Brassica juncea* L.). Plants were grown with/without Cu stress and treated during pre-germination for 3 h with water, SNP, FCy (SNP analogue) and SNP + cPTIO (cPTIO as a specific NO-scavenger). Control: **A** and **G**; **B** and **H**: Cu; **C** and **I**: SNP; **D** and **J**: SNP+Cu; **E** and **K**: FCy + Cu; **F** and **L**: SNP+cPTIO+Cu.

Copper induced H_2_O_2_ generation was visualized in roots by staining with H2DCFDA an indicator for ROS predominantly H_2_O_2_ in cells (Figure 9A–F). This reagent passively diffused into cells and its acetate groups cleaved by esterases. Upon oxidation, by H_2_O_2_ the non-fluorescent probe H2DCFDA gets converted to the highly fluorescent 2′,7-dichlorofluorescein (DCF). In this study, root cells of Cu stressed and FCy or SNP in combination with cPTIO-treated plants in the presence of Cu yielded higher intensity of green fluorescence while SNP reduced the effect of Cu stress and showed the less intensity of green fluorescence like that produced in control plants (Figure 9A–F).

Propidium iodide staining (an indicator of cell death) used to visualize cell viability by identifying the nucleic acid staining. Propidium iodide is membrane impairment and generally excluded from viable cells and in dead cells reaches the nucleus through distorted areas of dead cell membranes. In our study root cells of Cu stressed, FCy and of SNP in combination with cPTIO were less viable. However, Cu induced cell death was reduced by SNP application, showing similar response as was shown in control plants (Figure 9G–L).

### 3.10. Effect of NO on the Activity of Antioxidant Enzymes

**Figure 10:**
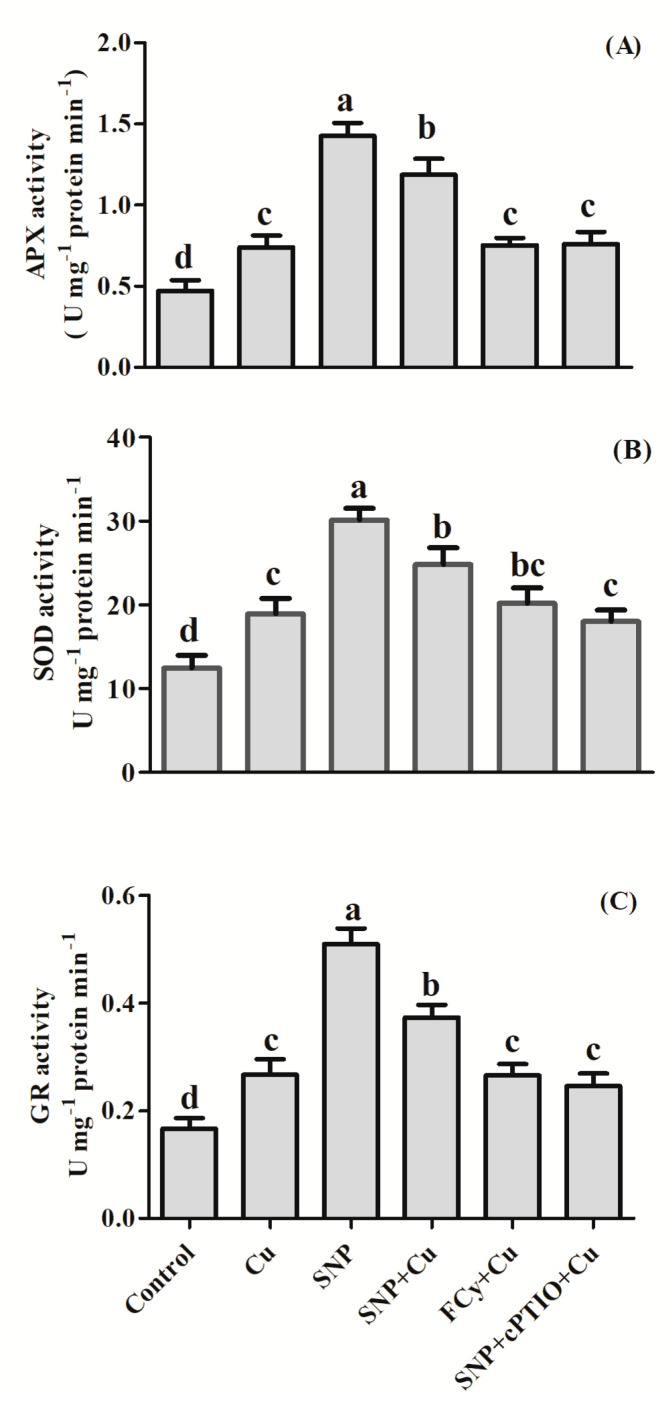
APX (**A**), SOD (**B**) and GR (**C**), activity in leaves of Indian mustard (*Brassica juncea* L.) at 30 DAG. Plants were grown with/without Cu stress and treated during pre-germination for 3 h with water, SNP, FCy (SNP analogue) and SNP + cPTIO (cPTIO as a specific NO-scavenger). Same letters above bars show that data did not differ significantly by LSD test at *p* < 0.05.

In Figure 10, it is apparent that in the leaves of *B. juncea*, SOD activity increased with Cu treatment in contrast to the control. Sodium nitroprusside enhanced SOD activity to a higher level than in the presence of Cu alone. However, pre-treatment of SNP in stressed plant maximally elevated SOD activity, which was by 99.6% when compared to the control. Potassium ferrocyanide (FCy) and cPTIO in combination with SNP did not alter the SOD enzyme activity in the stressed plant and showed a response comparable to the control.

Copper treatment increased the activity of GR and APX by 60.1% and 56.3%, respectively, as compared to control. Maximum increase in GR and APX activity was noted with the supplementation of NO (Cu + SNP), which increased the activity of both by about 40% and 60%, respectively, in leaves in comparison to the Cu-treated plant. The addition of NO-scavenger cPTIO nullified the influence of NO. Apart from that FCy also showed null effect on antioxidant enzymes activities in the Cu exposed plant.

### 3.11. Impact of NO on Photosynthetic Performance

Compared to the control plants getting NO in the form of SNP exhibited higher values for photosynthetic characteristics, such as P_N_, Ci, gs, maximal PSII photochemical efficiency, and Rubisco activity. Supplementation of NO in non-Cu fed plants improved P_N_ value by 57%, Ci by 49.3%, gs by 36.2%, maximal PSII photochemical efficiency by 33.3% and Rubisco enzyme activity by 35% when compared to the control. However, Cu treatment reduced the values of above parameters by 38%, 21.7%, 29.5%, 45.1% and 40.7%, respectively in comparison to control. Further, in Cu-treated plants, NO alleviated Cu induced reduction and showed increased values for P_N_, Ci, gs, and PSII photochemical efficiency and Rubisco enzyme activity by 114%, 74.8%, 84.4%, 114.7% and 89%, respectively, in comparison to the Cu treated plants. However, in case of supplementation of FCy the results were same for all photosynthetic parameters as shown under Cu alone condition. The treatment of cPTIO in combination with NO nullified the effect of NO, resulted in reduced photosynthetic parameters in Cu exposed plants (Table 2).

### 3.12. Effect of NO on Physiology of Guard Cells

**Figure 11:**
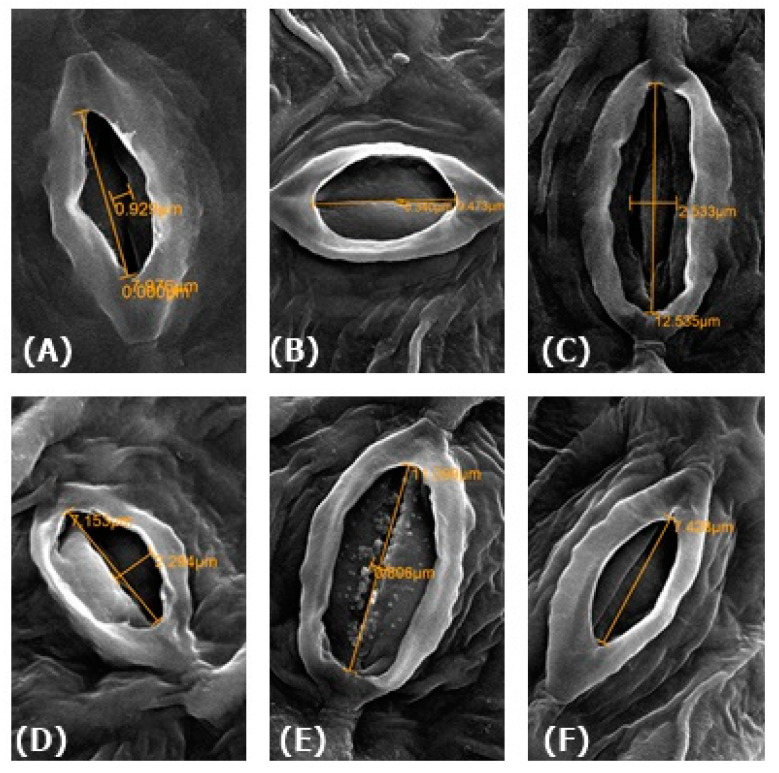
Stomatal response of Indian mustard (*Brassica juncea* L.) leaves at 30 DAG under control (**A**), Cu (**B**), SNP (**C**), SNP + Cu (**D**), FCy+ Cu (**E**) and SNP + Cu +cPTIO (**F**) at 4000× using scanning microscope.

Changes in stomatal structure in response to SNP, FCy and cPTIO were studied through electron microscopy. Stomatal analysis depicted a noteworthy change in the stomatal functioning in Cu exposed plants. Stomatal opening (length and width) was 7.96 and 0.92 μm on 30th DAG in control plants (Figure 11). The stomatal opening was about 9.42 μm in pore length and stomata were found closed in leaf samples of Cu-exposed plants. However, the supplementation of SNP in the presence of Cu showed an increase in the stomatal aperture by about 2.29 μm in diameter. Conversely, treatment of SNP applied plants resulted in the maximal stomatal opening of 2.53 μm and 12.54 μm in pore length and pore width respectively. Further, the application of SNP treated Cu-stressed plants with its scavenger cPTIO showed decrease in the stomatal aperture by about 0.81 μm in diameter. However, when FCy was applied to Cu-treated plants showed the same result as plants grown under Cu treatment alone (Figure 11A–F).

**Figure 12:**
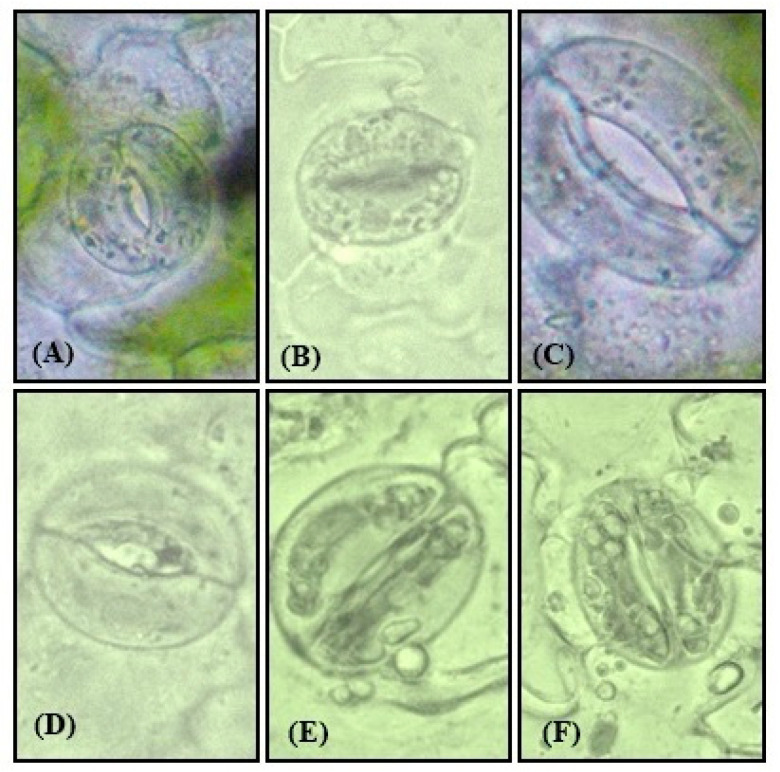
Stomatal response of Indian mustard (*Brassica juncea* L.) leaves at 30 DAG under control (**A**), Cu (**B**), SNP (**C**), SNP+Cu (**D**), FCy+Cu (**E**) and SNP+Cu+cPTIO (**F**) at 40× using compound microscope.

The assertions of electron microscopy were further expanded by observations under compound microscopy. Stomata in leaf samples of control were normal with specialized guard cells, SNP applied in Cu treated plants showed an increase in stomatal aperture as compared to closed with distorted guard cells in stressed plants (Figure 12A–F).

### 3.13. Effect of NO on NO Generation, GSH Content, Plant Dry Mass and Leaf Area

**Figure 13:**
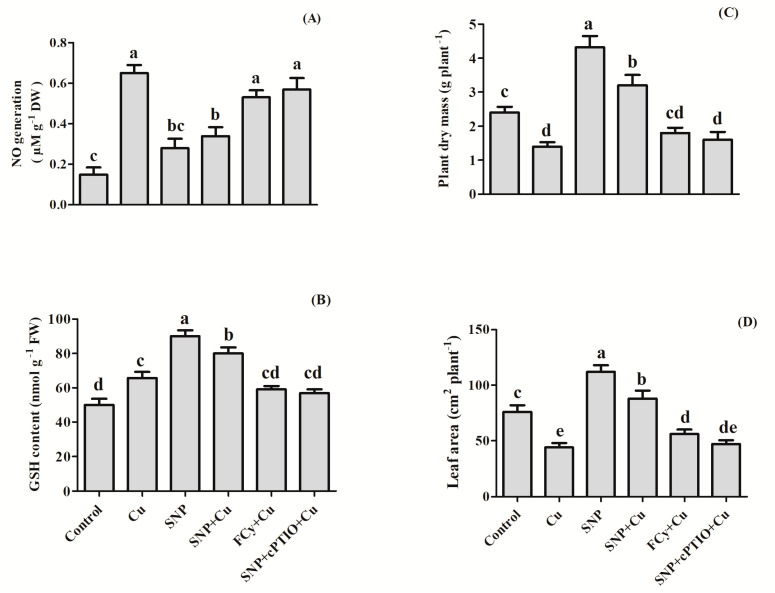
Effect of NO on NO generation (**A**)**,** GSH (**B**), plant dry mass (**C**) and leaf area (**D**) of Indian mustard (*Brassica juncea* L.) treated during pre-germination with water, 100 µM SNP or SNP with cPTIO and FCy in presence or absence of 3 mM Cu at 30 DAG. Same letter above bars show that data did not differ significantly by LSD test at *p* < 0.05. FW—fresh weight; DW—dry weight.

Figure 13 revealed that plants grown in Cu, exhibited increased NO generation by 4.3 times compared to control plants, but pre-germination supplementation of SNP decreased NO generation by 1.9 times compared to Cu-exposed plants. However, minimum decrease i.e., not much significant change in NO generation was noted by about 1.2 times with exogenously applied FCy. Furthermore, application of cPTIO in combination with NO resulted in the reversal of the outcome of NO generation in Cu-exposed plants.

*B. juncea* treated with 3.0 mM Cu increased GSH content while there was a reduction in plant dry mass and leaf area as compared to control. Plants with pre-germination treatment of SNP increased GSH content, and improved growth characteristics, plant dry mass and leaf area. Application of 100 µM SNP to Cu grown plants increased GSH content, plant dry mass and leaf area by 21%, 128%, and 100%, respectively as compared to Cu-treated plants, while the addition of cPTIO reversed the influence of exogenously applied NO and FCy application showed no significant change to Cu-exposed plants (Figure 13).

## 4. Discussion

Copper is an essential micronutrient required in a very low concentration by the plants for their proper growth and development and is a vital component of several biomolecules [6]. In excess of its permissible concentration, it has a high detrimental influence on seed germination and growth of plants [4,55]. In plants, NO acts as an important signaling molecule and has achieved an evident concern due to the fact of its crucial mitigating role in abiotic and biotic stresses [41,56,57,58].

In the current study, SNP as an efficient NO donor was used because it provides an absolute form of NO and supplies it for an extensively longer duration as compared to other NO donors [59,60]. However, SNP can also release cyanide (CN) and/or can form CN-Fe complex. It has been proposed that these complexes of CN may have an overlapping or distinct role with the role of NO alone on biological tissues [60]. Earlier, NO has been reported to play a significant role in the seed germination process in different plant species including warm-season grasses [35], *Arabidopsis* [34], and lettuce [32]. NO-mediated attenuation of the inhibition of germination of rice seed and the amelioration of the inhibition of seedling growth have also been found under cadmium (Cd) regimes [61]. These authors have also noticed a cPTIO-mediated reversal of the protective effect of exogenous supplementation of SNP. Besides, the incapability of potassium ferricyanide in the release NO like FCy and its antagonistic effects in contrast to SNP was also observed. In *Paulownia elongata*, SNP application was found involved in the breaking of seed dormancy and induction of seed germination [62]. Pre-soaking treatment also showed an advantageous effect on the attenuation of NaCl-induced inhibition of seed germination and growth of pakchoi radicles and plumules [63]. In agreement with the data published on other plants, our results proved the protective and stimulatory role of SNP pre-treatment on *B. juncea* seed germination. Conversely, the NO-scavenger cPTIO reversed the NO-induced seed germination under Cu stress, while its analogue FCy showed no change in germination percentage in comparison to Cu treated seeds. Results also confirmed that NO derived from SNP but not any other compound FCy was responsible for germination under Cu stress in mustard seeds. Additionally, the use of its analogue (FCy) did not work in mitigation but also inhibition of NO generation using cPTIO was proved ineffective. Sodium nitroprusside pre-treated *B. juncea* seeds were able to induce a rapid rise in the activity of amylase enzyme as compared to the stressed seeds with a steady rise to a maximum till 24 h (Figure 6). According to Zhang et al. [64], the amylase activity induced by NO was independent of gibberellic acid, showing that NO was involved in seed germination under various conditions. Similarly, Patel et al. [65] showed the involvement of NO in inducing germination of primed maize seeds.

Under Cu stress, *B. juncea* seeds not only showed inhibition in germination but also exhibited enhanced levels of TBARS and H_2_O_2_ content, even higher content was observed at the vegetative phase. These outcomes are in harmony with the earlier work of Hu et al. [36] and Fatma et al. [39], showing Cu and salt stress increased the accumulation of H_2_O_2_ and TBARS contents in wheat seeds and *B. juncea* leaves respectively. Per et al. [41] emphasized that NO tends to decrease the levels of TBARS and H_2_O_2_ content safeguarding the cell membrane to reduce the cell membrane damage through lipid peroxidation. An appropriate concentration of NO is required to induce an antioxidant system to prevent ROS induced oxidative damage that alleviates plant fitness loss [66]. In confronting heavy metal stress, NO was found to regulate the antioxidant system, including both the enzymatic and the non-enzymatic antioxidant system to alleviate ROS production [67]. This study further confirmed that the NO application reduced the production of ROS, H_2_O_2_, and O_2_^−^ using DAB and NBT staining methods in both Cu fed plants and germinating seeds. This may be because NO detoxifying ROS by forming new compound peroxynitrite when reacts directly with the O_2_^−^. This newly formed compound itself acts as a potential signaling molecule in stress response, and also functions in the regulation of protein activity [68]. It has also been reported that NO improves Cu-tolerance by regulating H_2_O_2_ and O_2_^−^ [69]. Furthermore, a plethora of literature depicts the involvement of NO in the inhibition of ROS accumulation of O_2_^−^ and H_2_O_2_ and their subsequent localization using NBT and DAB staining [70,71,72]. Nitric oxide application also exhibited an inhibitory effect on Cu uptake in both shoots and roots in Cu fed plants (Figure 7A,B). Our results are following the outcomes of Wang et al. [73] in tomato, Mostofa et al. [74] in rice and Zhang et al. [75] in tomato seedlings.

The present study also indicated the participation of NO in enhancing the activities of antioxidant enzymes including APX, GR, and SOD in pre-treated germinating seeds and leaves of *B. juncea* grown plants under Cu stressed condition. In compliance, various earlier published reports have proved the expression of antioxidant enzymes by the supplementation of NO in *Oryza sativa* under nickel toxicity [76], *Lycopersicon esculentum* under salinity stress [58], and in cotton under NaCl stress [77]. Cu-pretreatment exhibited several times increase in Cu-concentrations both in roots and leaves of plants. However, the accumulation in leaves was lesser compared to roots (Figure 7). Limiting heavy metal in roots has been thought to be a mechanism of plant heavy metal stress tolerance. In earlier studies, as a primary strategy of a plant to counter potential metal-toxicity, roots have been found as efficient barriers in the translocation of most of the metals to the aboveground plant parts [78,79,80,81,82]. Herein, NO-addition increased the accumulation of Cu in roots and thereby decreased its contents in shoots. This result indicated the NO-mediated inhibition of excess Cu transport toward shoots. Involvement of NO-mediated S-nitrosylation (a regulator of protein activation) and the actions of PCs and some other -SH containing thiols were argued to be involved in the detoxification of heavy metals [83]. Therefore, NO-induced low Cu concentration in *B. juncea* leaves contributed to the increase of Cu tolerance. It was also observed that cPTIO treatment for 48 h could induce Cu accumulation in Cu-stressed periwinkle seedlings [84]. From these results, it can be concluded that NO might play an important role in reducing the Cu accumulation in mustard plants. In this study, SNP application was found to significantly upregulated GR activity and thereby increased the reduced pool of GSH, a major constituent of metal-chelating PCs (Figure 10 and Figure 13). Mostafa et al. [74] also confirmed that NO and GSH co-treatment was more effective in reducing Cu accumulation than lone treatment of SNP.

It has also been concluded by various studies that NO could confront much of the oxidative damage by regulating the activity of antioxidant enzymes in plants under heavy metal stress [85,86,87]. Our results are in agreement with the above works where APX, SOD, and GR activity were induced under SNP pre-treatment. Nitric oxide proved to be more promising and impactful in increasing photosynthetic characteristics, PN, Ci, gs, Rubisco activity, and PSII photochemical efficiency in the presence and absence of Cu stress (Table 1). The mechanism underlying the positive influence of NO on photosynthesis under Cu-toxicity may be correlated with the increased activity of antioxidant enzymes, the protection of chlorophyll, and decreased ROS accumulation. Earlier, it has been suggested that the NO can control the photosynthetic efficiency rate by controlling the size of the stomatal aperture, hence influencing the stomatal conductance [39,88]. SNP application also has some positive role in improving the activity of the Rubisco enzyme and ultimately enhancing photosynthetic activity. The study is supported by numerous findings such as protection of chlorophyll damage and enhancement in photosynthetic pigments in NO-treated *Lolium perenne* under Cu stress [79], *Helianthus annuus* exposed to Cd stress [89], and *Triticum aestivum* [90] and *B. juncea* [39] under salt stress. Treatment of cucumber seedlings, with SNP, enhanced the chlorophyll content, rate of photosynthesis, and transpiration rate and stomatal conductance [91]. These results were mainly due to the enhancement of antioxidant machinery, prevention, and recovery of chlorophyll damage and increased GSH content. In our study, the effect of NO prominently increased the content of GSH in the absence and presence of Cu stress. The study of Per et al. [41] also showed that NO accelerated GSH production in mustard plants treated with Cd.

The application of SNP eased the effect of Cu stress on plant dry weight and leaf area (Figure 13). Foliar spray of NO on several crops protected against Cu toxicity in *Lycopersicon esculentum* [75], *B. juncea* [92], *Nicotiana tabaccum* [93] and also alleviated the effect of Ni stress in rice [76]. Growth improvement can also be associated with the escalation in antioxidant machinery after NO supplementation. The study of Bai et al. [94] in *L. perenne* revealed that the application of NO mitigated Pb stress, and alleviated negative effects on leaf growth by increasing the mineral nutrient and by differing the oxidative stress parameters. Maximum NO accumulation was observed in the Cu stressed plants (Figure 13). It is because excess Cu also induces nitrosative stress by excessive production of NO, which subsequently reacts with ROS such as O_2_, O_2_^−^ and H_2_O_2_ and results in other reactive nitrogen species in Cu stressed plants [95]. Thus, excessive accumulation of NO, along with H_2_O_2_ might be a potential mechanism for enhanced lipid peroxidation. Exogenously-sourced NO has been established to prevent the increase of oxidative damage and endogenous NO content in plants and increase tolerance of plants to Cu stress by up-regulation of the antioxidant system (APX, GR, and SOD) thereby ROS quenching which retained the cellular osmotic adjustment and protected photosynthetic machinery from Cu stress by acting as oxygen radical scrapper. SNP application resulted in the reduction of NO generation in plants grown with or without Cu stress. However, a minimum reduction of NO generation was found in Cu stressed plant and exogenously applied FCy as NO analogue which did not release NO on its breakdown in Cu stressed plant. Further, the application of cPTIO in combination with NO resulted in the reversal of the effect imposed by SNP on plants exposed to Cu. Wang et al. [96] also found that NO content increased in tomato plants in presence of Cu toxicity. We have earlier shown the mitigating effect of exogenous NO application under various abiotic stresses [39,41,50,88], where SNP was sprayed on the vegetative phase of the plants. In the following experiment, seeds were pretreated showing similar responses as procured in the above experiments, and the dose of SNP used was reduced to a much smaller level. Suggesting the use of pre-treatment of seeds could be a better strategy in improving the stress tolerance mechanism in plants with better germination percentage.

## 5. Conclusions

This study demonstrated that NO pretreatment was able to improve seed germination and reduced inhibitory effect of Cu by modulating antioxidant system, ROS accumulation, lipid peroxidation, and amylase activity. It also confirmed that pretreatment of NO significantly mitigated Cu toxicity also during the vegetative phase through improving the antioxidant system, photosynthetic efficiency, and reducing Cu induced accumulation of ROS accompanied by a reduction in lipid peroxidation of the mustard plant. Nitric oxide-pretreatment could be argued as a potential way of application that has shown here exhibiting both pre and post-germination-effects on *B. juncea* plants. From our results, it is concluded that no other compounds except NO derived from SNP, was able to promote seed germination and photosynthetic efficiency under Cu stress in Indian mustard. Therefore, pre-treatment of seeds with NO could be employed as a key biochemical approach for alleviating Cu-toxicity during seed germination, as it not only resulted in improving germination rate, but the applied NO also led to an improved vegetative phase of the plants.

## Figures and Tables

**Table 1:** Experimental design.

Treatments	Details
Control	Seeds pre-treated with water and raised without Cu stress
Cu	Seeds pre-treated with water and raised with Cu stress
SNP	Seeds pre-treated with SNP and raised without Cu stress
SNP + Cu	Seeds pre-treated with SNP and raised with Cu stress
FCy + Cu	Seeds pre-treated with FCy and raised with Cu stress
SNP + cPTIO + Cu	Seeds pre-treated with SNP+cPTIO and raised with Cu stress

Cu—Copper; SNP—Sodium nitroprusside; FCY—Potassium ferrocyanide; cPTIO—2-(4-carboxyphenyl)-4,4,5,5-tetramethylimidazoline-1-oxyl-3-oxide.

**Table 2:** Net photosynthesis, intercellular CO_2_ concentration, Maximal PSII photochemical efficiency, stomatal conductance, and Rubisco activity in Indian mustard (*Brassica juncea* L.) at 30 DAG. Seeds were treated at pre-germination time with water, 100 µM SNP or SNP with cPTIO and FCy in presence or absence of 3.0 mM Cu.

Treatments	Net Photosynthesis (µmol CO_2_ m^−2^ s^−1^)	Internal CO_2_ Concentration (µmol CO_2_ mol^−1^)	Maximal PSII Photochemical Efficiency	Stomatal Conductance (mmol CO_2_ m^−2^ s^−1^)	Rubisco Activity (µmol CO_2_ mg^1^ protein min^−1^)
Control	16.67 ± 0.89 ^c^	203 ± 5.57 ^c^	0.6 ± 0.09 ^c^	288 ± 9.54 ^b^	0.82 ± 0.06 ^b^
Cu	10.27 ± 0.92 ^e^	159 ± 5.51 ^d^	0.34 ± 0.06 ^d^	203 ± 8.54 ^c^	0.49 ± 0.05 ^c^
SNP	26.16 ± 0.72 ^a^	303 ± 4.04 ^a^	0.8 ± 0.08 ^a^	392.67 ± 8.19 ^a^	1.06 ± 0.07 ^a^
SNP + Cu	22 ± 0.58 ^b^	278 ± 6.08 ^b^	0.7 ± 0.06 ^b^	374.33 ± 8.68 ^a^	0.92 ± 0.09 ^ab^
FCy + Cu	12.96 ± 0.80 ^d^	168 ± 5.29 ^d^	0.38 ± 0.07 ^d^	221 ± 6.66 ^c^	0.52 ± 0.04 ^c^
SNP + cPTIO + Cu	11.97 ± 58 ^de^	162 ± 4.51 ^d^	0.35 ± 0.05 ^d^	208 ± 5.51 ^c^	0.51 ± 0.04 ^c^

The data followed by same superscripted letter are not significantly different by LSD test at *p* < 0.05.
